# Glutathione peroxidase (GPx) and superoxide dismutase (SOD) activity in patients with diabetes mellitus type 2 infected with Epstein-Barr virus

**DOI:** 10.1371/journal.pone.0230374

**Published:** 2020-03-25

**Authors:** Jakub Dworzański, Małgorzata Strycharz-Dudziak, Ewa Kliszczewska, Małgorzata Kiełczykowska, Anna Dworzańska, Bartłomiej Drop, Małgorzata Polz-Dacewicz

**Affiliations:** 1 Masovian Specialist Hospital, Radom, Poland; 2 Chair and Department of Conservative Dentistry with Endodontics, Medical University of Lublin, Lublin, Poland; 3 Department of Virology, Medical University of Lublin, Lublin, Poland; 4 Department of Medical Chemistry, Medical University of Lublin, Lublin, Poland; 5 Department of Information Technology and Medical Statistics, Medical University of Lublin, Lublin, Poland; University of North Carolina at Chapel Hill, UNITED STATES

## Abstract

Oxidative stress is suggested to be the crucial factor in diabetes mellitus type 2 (DM2) pathogenesis and in the development of diabetic complications. Patients with DM2 may be more susceptible to infections due to hyperglycaemia-induced virulence of various microorganisms. Several studies pointed that Epstein-Barr virus (EBV) infection is associated with reactive oxygen species (ROS) production and/or activation of signalling pathways connected with ROS. The present study analyzed serum activity of glutathione peroxidase (GPx) and superoxide dismutase (SOD) in DM2 patients with and without EBV infection. Blood and saliva were collected from 120 patients with DM2. EBV DNA was detected in the saliva using nested-PCR technique. Spectrophotometric methods were implemented to determine serum GPx and SOD activity with the use of diagnostic kits produced by Randox Laboratories. GPx and SOD activity was decreased in diabetic patients, with the lowest values in DM2 EBV-positive patients. There was correlation between GPx and SOD activity–with increased value of GPx, SOD activity was also rised. In patients with DM2 history longer than 10 years as well as in DM2 patients with obesity, antioxidant enzymes activity was decreased. Determination of examined parameters may be useful in diabetic patients with EBV infection and could be important prognostic factor.

## Introduction

Diabetes mellitus type 2 (DM2) has become one of the most important global health problems recently. It reaches epidemic proportions as in the year 2015 there were nearly 415 million people with diagnosed DM2 and by the year 2040 this number is expected to increase to 642 million [1,2].

Many reports suggest that oxidative stress is implicated in the pathogenesis of DM2 [3–6]. There are three major enzymes in antioxidant defense system: glutathione peroxidase (GPx), superoxide dismutase (SOD), and catalase (CAT) [7–8].

Epstein-Barr virus (EBV) is widespread human virus belonging to *Herpesviridae* family—more than 90% of adults are EBV-seropositive worldwide. As the first known human carcinogenic virus it is associated with various malignancies, such as Hodgkin's lymphomas (HL), Burkitt's lymphomas (BL), nasopharyngeal cancer (NPC) and gastric cancer (GC) [9]. EBV may establish a persistent infection and reactivate periodically into the lytic cycle which has impact on the pathogenesis of EBV-associated tumours [10–12]. According to some researches, patients with diabetes mellitus type 2 are more susceptible to infections due to hyperglycaemia-induced virulence of various microorganisms [13]. Other studies pointed that infection with EBV is associated with reactive oxygen species (ROS) production and/or with activation of signalling pathways connected with ROS [14,15].

Our previous research revealed that the prevalence of EBV DNA in patients with diabetes is significantly higher than in individuals without this disease [16]. The present study analyzed the serum activity of GPx and SOD in the patients with DM2 with and without EBV infection.

## Materials and methods

### Patients

The present study involved 120 patients with diabetes mellitus type 2 hospitalized at the Internal Division of the Masovian Specialist Hospital in Radom, Poland. The control group comprised 50 individuals without diabetes (patients treated at the same department due to other diseases than DM2) suitably selected in terms of sex, age, place of residence, smoking and alcohol consumption. All persons from the control group were EBV negative. In terms of socio-demographic features, smoking and alcohol consumption these groups did not differ and therefore the features did not affect the values of examined parameters.

The study was approved by the Medical University of Lublin Ethics Committee, and is in accordance with the GCP regulations (No. KE-0254/135/2017, 25 May 2017). Informed written consent was collected from all participants.

### Clinical specimens

Blood and saliva were collected from the patients and controls. EBV DNA was detected in the saliva. Oxidant parameters were detected in serum.

#### Saliva collection

About 5 ml of non-stimulated whole saliva was collected. The saliva samples were centrifuged at 1500 rpm at room temperature for 10 min, and then diluted (1:1) in PBS and frozen at -80°C until their analysis.

#### Serum collection

Venous blood samples from both the patients and the controls were centrifuged at 1500 rpm at room temperature for 15 min, followed by serum collection and frozen at -80°C until its analysis.

### Molecular methods

#### DNA extraction from saliva

DNA isolation was performed using the QIAamp DNA Mini Kit (Qiagen, Hilden, Germany) according to the manufacturer’s instructions. The efficiency and purity of the obtained eluate were checked using the Epoch (Biotek) spectrophotometer. The measurement was performed on a Take 3 plate (Biotek Instruments, Winooski, Vermont, US) using Microplate Reader Software Gen 5.2.0 (Biotek Instruments, Winooski, Vermont, US).

#### EBV DNA detection

EBV DNA detection and the amplification of the Epstein–Barr nuclear antigen 2 (EBNA-2) gene (the nested PCR) were performed as previously described [17]. The nested PCR was carried out for amplification of Epstein-Barr nuclear antigen 2 (EBNA-2). The sequence of primers used for PCR was as follows: outer pair 5’–TTT CAC CAA TAC ATG ACC C– 3’, 5’–TGG CAA AGT GCT GAG AGC AA– 3’ and inner pair 5’–CAA TAC ATG AAC CRG AGT CC– 3’, 5’–AAG TGC TGA GAG CAA GGC MC– 3’.

All PCR reactions were carried out in the final volume of 25 μl using HotStartTaq DNA Polymerase (Qiagen, Germany). Concentrations of PCR reaction components were prepared as follows: 2.0 mM MgCl_2_, 0.2 mMdNTPs, 0.5 μM of each forward and reverse primers and 0.5 U of HotStartTaq polymerase. During each run the samples were tested together with one negative (nuclease-free water) and positive control (EBV-positive cell line, Namalwa, ATCC-CRL-1432) [18].

#### Oxidant parameters

SOD activity was determined using diagnostic kit RANSOD produced by RANDOX (Randox Laboratories Ltd., Crumlin, Country Antrim, UK) according to Arthur and Boyne [19] and expressed in U of SOD/10mgof protein.

GPx activity was determined using diagnostic kit RANSEL produced by RANDOX (Randox Laboratories Ltd., Crumlin, Country Antrim, UK) according to Paglia and Valentine [20] and expressed in U of GPx/mg of protein. Protein was measured using method of Bradford [21]. The assays were performed with the use of spectrophotometer SPECORD M40 (Carl Zeiss, Jena, Germany).

### Statistical analysis

Statistical analysis was performed using Pearson’s chi-square test, Fisher’s exact test for small groups, ANOVA Kruskal-Wallis test with post-hoc Dunn`s test and Spearman`s Rank Test. The statistical significance was defined as p < 0.05.

## Results

Clinical and epidemiological characteristics of the patients is presented in Table 1.

**Table 1:** Epidemiological characteristics of EBV-positive and EBV-negative diabetic patients.

	EBV-positive N = 60		EBV-negative N = 60		p
	N	%	N	%	
**Sex**					
Male	25	42.7	24	40.0	>0.05
Female	35	58.3	36	60.0	
**Age**					
20–39	8	13.3	8	13.3	>0.05
40–59	22	36.7	22	36.7	
60+	30	50.0	30	50.0	
**Place of residence**					
Urban	25	41.7	24	40.0	>0.05
Rural	35	58.3	36	60.0	
**Smoking**					
Yes	36	60.0	38	63.3	>0.05
No	24	40.0	22	36.7	
**Alcohol abuse**					
Yes	35	41.1	36	60.0	>0.05
No	25	58.3	24	40.0	
**BMI**					
18.5–24.9	10	16.7	11	18.3	>0.05
25–29.9	15	25.0	16	26.7	
30–39.9	35	58.3	33	55.0	
**Duration of diabetes (years)**					
1–5	14	23.3	13	21.7	>0.05
6–10	15	25.0	15	25.0	
>10	31	51.7	32	53.3	

BMI–body mass index, N–number of patients (Pearson`s chi-square test)

**Fig 1:**
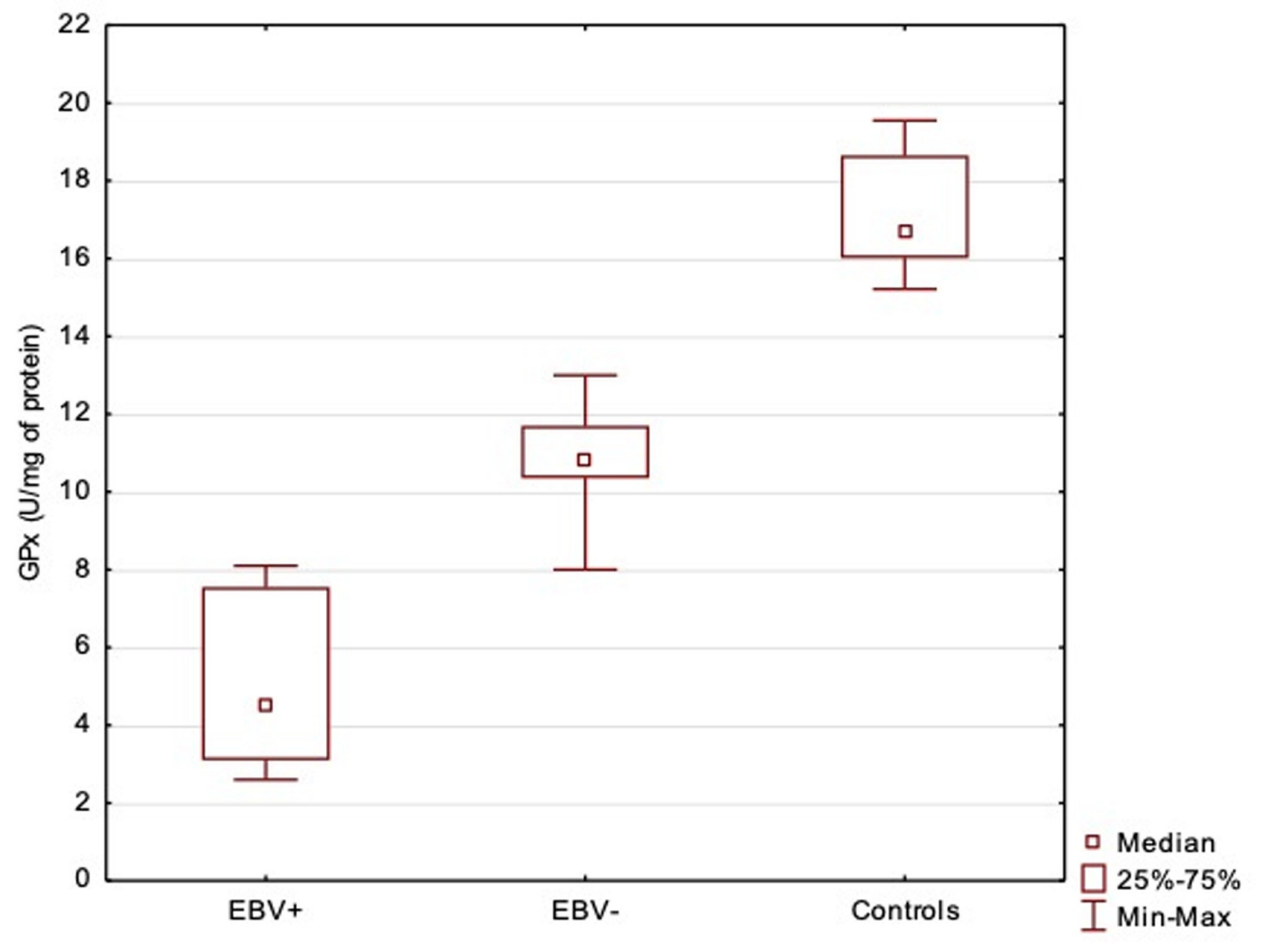
Serum activity of GPx in EBV-positive, EBV-negative diabetic patients and control group. ANOVA Kruskal-Wallis Test (H = 128.22; p < 0.0001); Post-hoc Dunn’s Test: EBV+/EBV-p< 0.0001; EBV+/Control p< 0.0001; EBV-/Control p< 0.0001.

**Fig 2:**
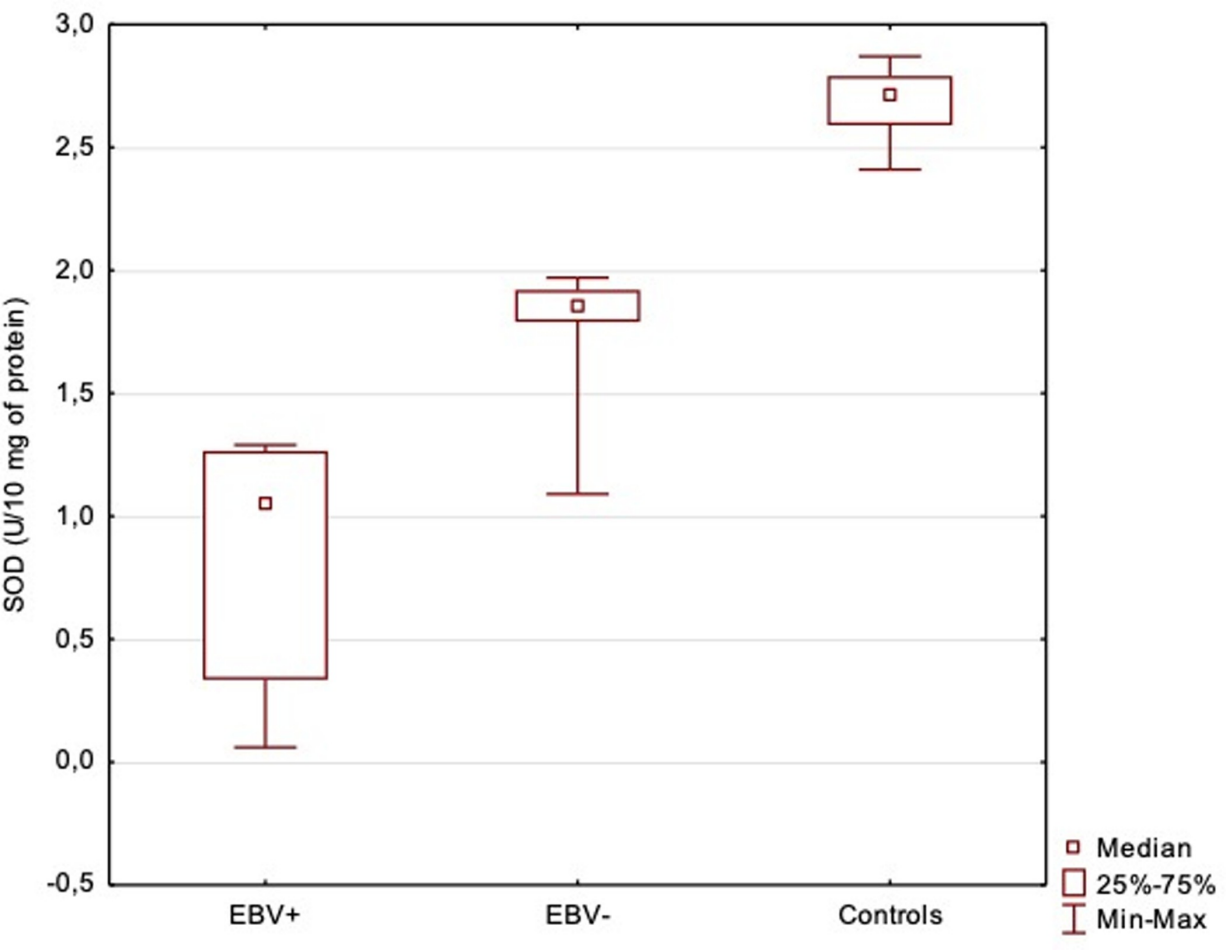
Serum activity of SOD in EBV-positive, EBV-negative diabetic patients and control group. ANOVA Kruskal-Wallis Test (H = 126.92; p < 0.0001; Post-hoc Dunn’s Test: EBV+/EBV- p< 0.0001; EBV+/Control p< 0.0001; EBV-/Control p< 0.0001.

GPx and SOD activity in the serum of patients with DM2 was statistically lower than in control group. Statistically significant differences were stated in GPx and SOD activity in EBV-positive and EBV-negative patients (Figs 1 and 2). Both analysed parameters had the lowest activity in EBV-positive patients (GPx = 5.18±2.19, SOD = 0.88±0.45) and the highest in the control group (GPx = 17.23 ± 1.34, SOD = 2.68±0.14).

**Fig 3:**
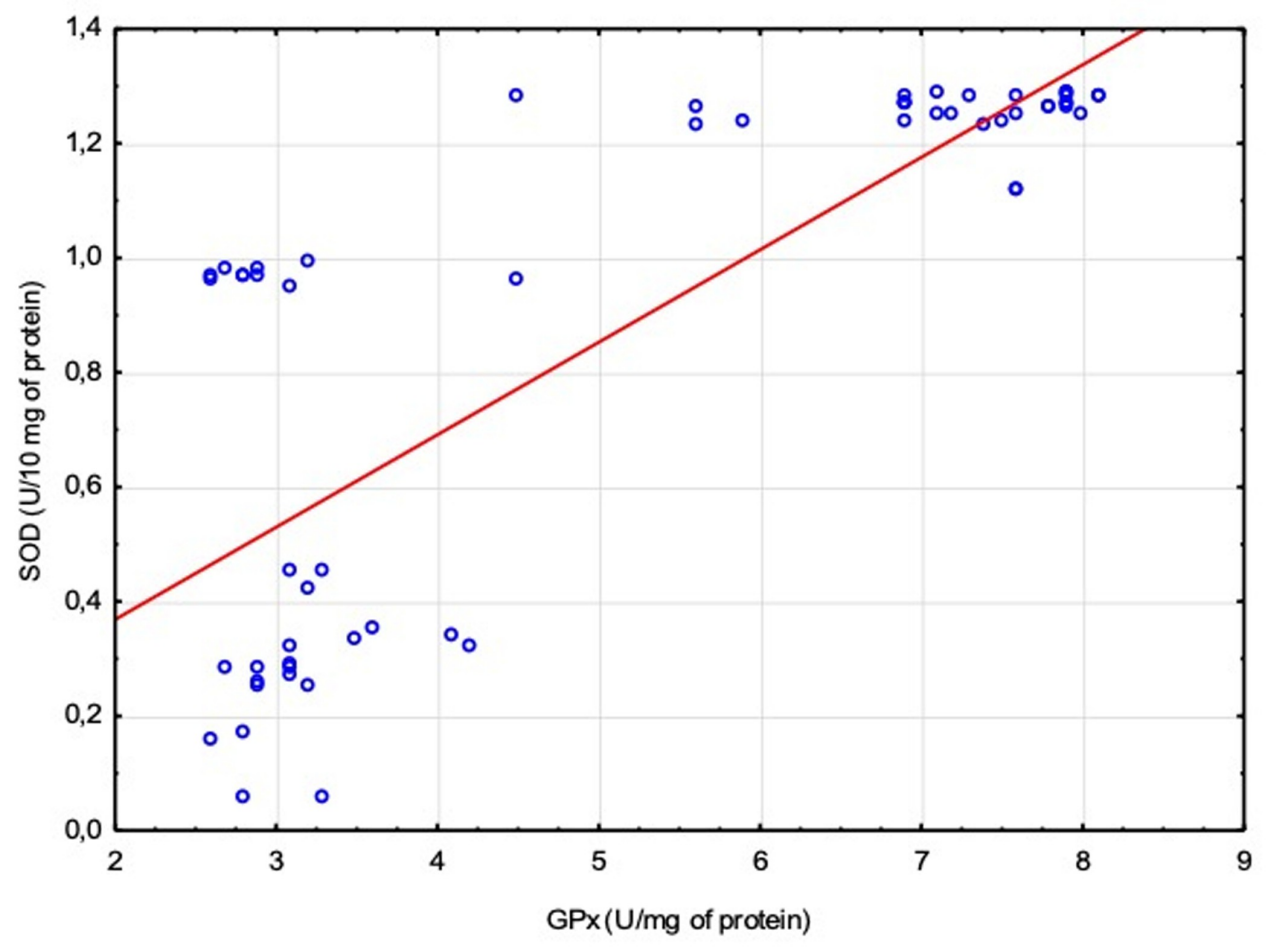
Correlation between GPx and SOD activity in EBV-positive diabetic patients. Spearman’s Rank Test (R = 0.7784; p < 0.0001).

Fig 3 shows correlation between serum activity of GPx and SOD in EBV-positive diabetic patients. With increased activity of GPx there was also increase in the activity of SOD.

Activity of antioxidant enzymes depended on the duration of diabetes and BMI index (Table 2). Activity of both GPx and SOD were significantly lower in patients with a DM2 history longer than 10 years (GPx = 3.10; SOD = 0.43) and in diabetic patients with obesity (GPx = 3.10; SOD = 0.43).

**Table 2:** GPx (U/mg of protein) and SOD (U/10mg of protein) activity according to duration of diabetes and BMI in EBV-positive diabetic patients.

	GPx $\overset{-}{X}\pm SD$	p	SOD $\overset{-}{X}\pm SD$	p
**Duration of diabetes (years)**				
0–5	7.39±0.63	<0.0001	1.26±0.03	<0.0001
6–10	4.10±1.65		0.95±0.36	
>10	3.10±0.41		0.43±0.29	
**BMI**				
Normal	7.40±0.62	<0.001	1.25±0.04	<0.001
Overweight	3.66±1.07		0.92±0.37	
Obesity	3.10±0.41		0.43±0.29	

BMI–body mass index, GPx—glutathione peroxidase, SOD—superoxide dismutase; ANOVA Kruskal-Wallis Test; GPx H = 44.48; p < 0.0001; SOD H = 42.87; p < 0.0001.

## Discussion

According to various studies oxidative stress is the crucial factor in DM2 pathogenesis as well as in the development of diabetic complications [3,5,22,23].

Although diabetes is associated with an increased production of ROS, the reports about the antioxidant defense in diabetes are not conclusive. Our study revealed decreased activity of GPx and SOD in patients with DM2 in comparison with control group. These findings are in agreement with other researches outcomes [5,8,24–28]. Lowered activity of these enzymes might be explained by the depletion of antioxidant defense system following the overgeneration of free radicals [29,30]. In diabetic patients glucose autooxidation results in formation of hydrogen peroxide which inactivates SOD. Accumulation of hydrogen peroxide may be one of the explanations for decreased activity of this enzyme [28]. GPx in normal conditions is a relatively stable enzyme, while in severe oxidative stress conditions it may be inactivated. Additionally, high glucose condition may cause inactivation of this enzyme by its glycation [28]. Moreover, hyperglycaemia and overproduction of ROS depress the endogenous antioxidant system and expose cells to damage due to oxidative stress which can initiate diabetic complications [23].

Some studies demonstrated, however, increase in SOD and GPx activity in patients with DM when compared with controls, despite the presence of oxidative stress [31,32]. Al-Rawi [33] reported increased activity of SOD both in saliva and in serum of patients with DM2, while Aouacheri et al. [34] revealed increase in SOD value with lowered GPx activity in diabetic patients. Decreased activity of these enzymes was also stated in our study in patients with longer duration of the disease. It is in line with the results obtained by Briggs et al. [8], as they revealed significant decrease in the level of antioxidant enzymes level with increasing years of the disease.

Singh et al. [28], who performed studies in diabetic patients with and without complications, reported the highest SOD and GPx activity in non-diabetic individuals, lower in diabetic patients without complication and the lowest in DM2 patients with complications. Analysis of antioxidant enzymes activity in DM2 diabetic patients regarding EBV infection carried out in our study revealed a similar tendency–the values of GPx and SOD was the highest in the individuals without diabetes, lower in EBV(-) patients and the lowest in EBV(+) patients. According to our researches both EBV infection and diabetes are associated with lower values of antioxidant enzymes.

Our previous studies revealed that EBV DNA was detected more frequently in the group of diabetic patients (35.9%) than in patients without diabetes [16]. Chronic infections caused by various viruses influence the whole immunity of the patient. However, there are few reports in the literature about the relationship between DM2 and viral infections. EBV is also a proven risk factor for various malignancies, but no studies evaluating the relationships between viral infection, oxidative stress and diabetes are available. Some researches point to connection between infection with EBV and oxidative stress, especially in EBV(+) tumours [35]. It was demonstrated that oxidative stress and factors leading to DNA damage induce the expression of EBV lytic genes. As it was also suggested, the virus in the process of EBV reactivation encodes several anti-apoptotic proteins, which may inhibit apoptosis. Therefore, reactivation of EBV is a substantial risk factor for disease related to EBV [36].

There are reports about malignancies developing more frequently in diabetic patients, especially in individuals with DM2 [37]. Our previous study revealed that in cancer patients with EBV infection there was decrease in the value of antioxidant enzymes [38]. The present research showed that in diabetic patients infected with EBV the activity of SOD and GPx was also lowered. As EBV is a proven oncogenic virus, diabetic patients infected with EBV may be at higher risk of development of malignant diseases. In our research only 6% of diabetic patients were diagnosed with cancer, so the group was too small for statistical analysis, but this matter may be the subject of our future studies. Determination of these parameters may be useful in EBV(+) DM2 patients and could be an important prognostic factor as well as a marker of diabetic complications.

## Conclusions

Activity of SOD and GPx was decreased in diabetic patients, with the lowest values in EBV(+) patients with DM2. There was correlation between GPx and SOD activity–with increased activity of GPx there was also increase in the activity of SOD. In patients with DM2 history longer than 10 years and obesity, the activity of antioxidant enzymes was decreased.
